# The climate crisis is a health crisis: A Statement by Nordic Doctors for Planetary Health and Climate Action

**DOI:** 10.1177/14034948241302923

**Published:** 2025-03-13

**Authors:** Ola Løkken Nordrum, Ingrid Mobacke, Tuija MäNnistö, Martin Schønemann-Lund, Hjalti Már Björnsson, Lisbet Sviland

**Affiliations:** 1 Irish Doctors for the Environment; 2 Department of Anaesthesiology, St James’s Hospital, Dublin, Ireland; 3 Swedish Doctors for the Environment, Gävle, Sweden; 4 Gavle Hospital, Gavle, Sweden; 5 Lääkärin Sosiaalinen Vastuu, Kuopio, Finland; 6 University of Eastern Finland, Institute of Clinical Medicine; 7 Læger for Klimaet, Copenhagen, Denmark; 8 Emergency Department, Landspitali, Reykjavík, Iceland; 9 Icelandic Society of Doctors for the Environment; 10 Legenes klimaaksjon, Oslo, Norway; 11 University of Bergen, Faculty of Medicine and Dentistry, Bergen, Norway

In 1993, the Norwegian physician Per Fugelli wrote ‘the patient Earth is sick’ [1]. Over three decades later, the state of the Earth continues to deteriorate. We have already exceeded many safe Earth system boundaries [2]—our life-support systems. Our planet has developed multi-organ failure on our watch. Yet, there is hope, but we must act now.

This statement, co-written by doctors from Norway, Sweden, Denmark, Finland, Iceland and Ireland is our attempt to motivate both our own medical communities and our respective governments to act.

In 2009, *The Lancet* declared climate change the biggest threat for human health of the 21st century [3]. In 2023, over 200 medical journals identified climate change and the loss of biodiversity as a health crisis [4]. Despite these efforts, awareness of the impact of climate change on human health and the efforts to reduce healthcare emissions remains limited. Current efforts to mitigate the climate and biodiversity crisis need to be multiplied and the healthcare sector must participate in these activities on all levels.

The annual mean temperature in 2023 was 1.45°C above pre-industrial levels, making it the hottest year recorded since measurements began [5]. The environmental effects of this rise in temperature are already causing thousands of excess deaths [6]. The vast majority of Intergovernmental Panel on Climate Change climate scientists do not believe we will stay below the 1.5°C mean temperature target [7].

We urge people not to give up. The effects of the climate crisis will be one of the major challenges facing the healthcare sector. We need to recognize this and act now to ensure we are properly prepared. As health professionals, we recognise that the transition to a 1.5-degree lifestyle will affect material wealth. However, the green transition has the possibility to greatly improve public health through reducing air pollution, adopting a mainly plant-based diet and increasing physically active transportation. As healthcare workers, we must not only work to reduce emissions from our healthcare services, but also be a voice of science, informing the general public not only about the dangers of the climate and biodiversity crisis, but also the endless positives associated with taking action.

We call on our medical bodies and on our governments to act:

1. **Fossil Fuels:** A rapid fossil fuel phaseout is necessary to stop further global warming and harm to human health. Our national medical associations must join the World Health Organisation and the World Medical Associations in signing the Fossil Fuel Non-Proliferation treaty.
2. **Health Crisis:** Our respective medical bodies and governments should declare the climate and biodiversity crisis a health crisis. Healthcare workers should be a clear voice of science and educate the general public on the negative health effects of the climate and biodiversity crisis, but also the endless positives associated with taking action.
3. **Sustainable Healthcare:** Greenhouse gas emissions from the health sector must be reduced by at least 50% by 2030. Sustainable healthcare and planetary health must become key components of medical education.
